# Mayer–Rokitansky–Küster–Hauser (MRKH) syndrome with unilateral pulmonary agenesis—a rarity indeed: radiologic review

**DOI:** 10.1259/bjrcr.20150157

**Published:** 2016-02-03

**Authors:** Shivakumar Swamy Shivalingappa, Surekha Bhujanga Shetty

**Affiliations:** ^1^ Department of Radiology and Molecular Imaging, Health Care Global Enterprises, Bangalore, India; ^2^ Karnataka Institute of Diabetology, Bangalore, India

## Abstract

Mayer–Rokitansky–Küster–Hauser (MRKH) syndrome is a congenital condition characterized by agenesis of the uterus and vagina in females with normal ovaries and fallopian tubes, secondary sexual characteristics and 46XX karyotype. They present with primary amenorrhoea. Urinary anomalies, usually renal agenesis and rarely ectopia, occur. Skeletal abnormality can co-exist in about 10% of the patients. Simultaneous pulmonary hypoplasia has been reported very rarely in the literature. The normal external appearance of MRKH syndrome makes it difficult to diagnose until puberty. The purpose of this pictorial essay is to display the structural malformations of this rare disease. The presence of unilateral pulmonary agenesis is extremely rare. The use of invasive diagnostic laparoscopy and ionizing radiation, including intravenous urography or CT scan, has been reported in the literature for diagnosing MRKH. MRI is the mainstay of imaging in evaluating this syndrome, as it is free of radiation, non-invasive and has multiplanar capabilities.

## Case report

A 43-year-old female presented with a history of right-sided chest pain. She has been diabetic for the past 4 years and hypertensive, and has been on treatment. She had a history of primary amenorrhoea, which was never investigated previously. On examination, the patient was 155 cm tall, weighed 58 kg, blood pressure and secondary sexual characteristics were normal, and systemic examination showed absent breath sounds on the right side with mediastinal shift to the right.

## Radiological findings

Based on the initial clinical examination, a chest radiograph was performed. It showed complete opacification of the right hemithorax, with tracheal and mediastinal shift to the right. This prompted us to further evaluate with a CT scan of the chest and staged *T*_2_ weighted screening MRI of the spine, abdomen and pelvis.

**Figure 1.**
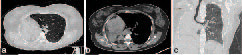
Axial non-contrast CT scans of the chest in the lung and mediastinal windows (a and b) show agenesis of the right lung. The left lung is hyperinflated. The mediastinum and the heart are displaced to the right. Coronal CT image in the lung window (c) also shows the same. The trachea continues as the left bronchus.

The CT scan of the chest revealed agenesis of the right lung with hyperinflation of the left lung. The heart and mediastinum had shifted to the right side (Figure 1). The trachea continued as the left main bronchus. No rudimentary bud was identified.

**Figure 2.**
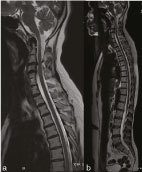
(a) Mid-sagittal *T*_2_ weighted sequence of the spine (cervical and whole spine) shows block vertebra at C4–C5. (b) Rest of the spine is normal with normal signal intensity of the spinal cord.

**Figure 3.**
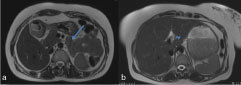
Serial axial *T*_2_ weighted sequence of the abdomen. (a) At the level of the pancreas, only the head and uncinate process are seen. The pancreatic tail and body are not seen in the normal anatomical position (arrow). Small bowel loops are seen in the pancreatic fossa. (b) The pancreatic body is seen extending cranially towards the liver hilum. The normal pancreatic duct can be seen (arrowhead).

**Figure 4.**
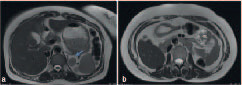
(a) The adrenal glands are normal. Incidentally seen is a small splenunculus (arrow). (b) The kidneys are not seen in the renal fossa.

**Figure 5.**
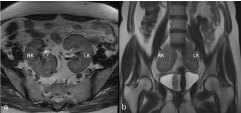
(a) Axial and (b) coronal *T*_2_ weighted images. Un-ascended pelvic kidneys are seen at the level of the sacral ala and the pelvic brim. LK, left kidney; RK, right kidney.

**Figure 6.**
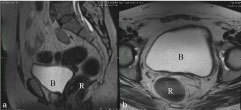
(a and b) *T*_2_ weighted mid-sagittal and axial pelvic images. The uterus is absent. B, urinary bladder; R, rectum.

**Figure 7.**
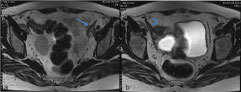
Axial weighted images of the pelvis. Ovaries are normal for the age. (a) Straight arrow, left ovary. (b) Curved arrow, right ovary.

**Figure 8.**
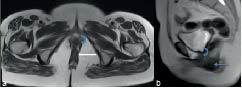
(a) Axial *T*_2_ weighted below the level of the pelvic floor and (b) mid-sagittal pelvic images. The vagina is absent. Straight arrows, anal canal; curved arrows, urethra.

A screening MRI of the spine showed block vertebra at the C4–C5 level (Figure 2). Axial *T*_2_ weighted sequence of the abdomen showed absent pancreatic tail and body in the normal anatomical position (Figure 3). The pancreatic body was seen extending cranially towards the liver hilum (Figure 3). The adrenal glands appeared normal (Figure 4). There were unascended pelvic kidneys bilaterally (Figure 5). The uterus was absent (Figure 6). The ovaries were seen, although they were slightly atrophic, considering the age of the patient (Figure 7). There was complete absence of the vagina on the MRI. Only the urethra was visualized (Figure 8).

## Other Investigations

As the patient presented with chest pain, an electrocardiogram was performed and found to be normal. Echocardio-graphy revealed situs solitus with dextroposition. Sclerotic aortic valve with trivial aortic regurgitation was seen. The left ventricular function was normal and no regional wall abnormalities were noted. The chest pain was concluded to be musculoskeletal in origin.

## Discussion

Mayer–Rokitansky–Küster–Hauser (MRKH) syndrome refers to a rare congenital condition characterized by aplasia of the uterus and the upper part (two-third) of the vagina in females with normal ovaries and fallopian tubes, secondary sexual characteristics and 46XX karyotype. MRKH syndrome is classified into two types based on associated anatomical features:

- Type I (isolated) MRKH syndrome: there is isolated uterovaginal aplasia and is referred to as Rokitansky sequence.
- Type II MRKH syndrome or MURCS (Müllerian duct aplasia, renal aplasia and cervicothoracic somite dysplasia) association: there is incomplete aplasia associated with other malformations including renal (unilateral agenesis, ectopia of the kidneys or horseshoe kidney), skeletal (Klippel–Feil anomaly or fused vertebrae, mainly cervical; scoliosis), hearing defects, cardiac and digital anomalies (syndactyly and polydactyly). It is referred to as MURCS association or genital renal ear syndrome.

MRKH syndrome occurs as a result of interrupted embryonic development and consequent midline fusion of the Müllerian ducts, which normally differentiate into fallopian tubes, uterus, cervix and upper part of the vagina. It occurs between the fifth and the sixth week of pregnancy. Primary damage to the mesoderm (paraxial, intermediate and lateral) is held responsible for skeletal, pulmonary, renal and Müllerian dysplasias present in MRKH syndrome.

The incidence of MRKH syndrome has been estimated as 1 in 4500 female births. The majority of cases appear to be sporadic, but few may be familial. It is an autosomal dominant condition, with incomplete degree of penetrance and variable expressivity. Type I (isolated) MRKH is less frequent than MURCS association.

Patients usually present with primary amenorrhea; normal secondary female sexual characteristics, external genitalia and 46XX karyotype; and normal and functioning ovaries with no signs of androgen excess.^1^ Apart from the gynaecological complications, females with MRKH syndrome have renal, skeletal, hearing or cardiac anomalies and increased levels of psychological distress. In summary, MRKH syndrome is a disabling pathology with anatomical, physiological and psychological profile.

MURCS association is a rare condition, reported infrequently in the literature. Oppelt et al^2^ has described 53 cases of MRKH syndrome in three subtypes: typical, atypical and MURCS association. Of the 521 cases included in the revision of the literature, 64% were typical, 24% atypical and only 12% MURCS. Malformations of the renal system were the most frequent type of accompanying malformation, seen in 19 patients, followed by 19 different skeletal changes in 15 patients of the Oppelt et al^2^ cases. In the Oppelt et al^2^ cases, there was no report of pulmonary agenesis. Renal malformations are reported in about 40–60% of cases.^3^ Simultaneous pulmonary anamolies, renal agenesis or dysplasia, and MRKH syndrome has been reported very rarely in literature; Acién et al^4^ have reported one case with pulmonary hypolplasia and unilateral renal agenesis. The common types of renal anomalies may include renal agenesis and ectopic pelvic kidney.^5^^–^^7^ Bachh et al^8^ reported a case of MRKH with unilateral pulmonary agenesis. Their case also had right lung agenesis with a rudimentary right bronchus (shown as a blind-ending pouch on the CT scan) and right renal agenesis with ectopic pelvic left kidney. In our case, there was no rudimentary bronchus on the CT images. Schneider and Schwalbe^9^ classified pulmonary agenesis into three groups, which were later modified by Boyden.^10^ Type I (agenesis) is a complete absence of the lung and the bronchus and there is no vascular supply to the affected side. In Type II (aplasia) there is a rudimentary bronchus with complete absence of the pulmonary parenchyma, and in Type III (hypoplasia) variable amounts of the bronchial tree, pulmonary parenchyma and supporting vasculature are present.

Our reported case can be classified as Type II pulmonary agenesis or aplasia. In this scenario, CT scan is an excellent tool to evaluate the tracheobronchial tree and lungs. With the advent of multislice CT scanner, high resolution thinner sections can be obtained. Virtual bronchoscopy data can be obtained from the same datasets to assess the airway. But, CT scan is not useful in evaluation of the pelvic organs owing to poor contrast resolution of the soft tissues. MRI on the other hand is a very good non-invasive method of assessing the pelvic organs. Uterine agenesis or hypoplasia is best diagnosed on *T*_2_ weighted multiplanar MRI images because of very high soft tissue resolution and multiplanar capabilities. *T*_2_ weighted sequences produce excellent zonal anatomy of the uterus, including the endometrium, junctional zone and myometrial anatomy. MRI also provides comprehensive imaging of the osseous and soft tissues. The spinal cord, disc and osseous structures can be imaged in a single setting. MRI is the only tool available to visualize the spinal cord and nerve roots and can provide an excellent intrinsic contrast resolution.

## Conclusions

The diagnosis of MRKH syndrome is usually made on the basis of clinical findings, but radiological evaluation is essential for confirmation. MRI is the imaging modality of choice, considering the ability to identify associated renal and skeletal anomalies. It is non-invasive and there is no use of ionizing radiation. It also has multiplanar capabilities, which makes it feasible in evaluation. CT scan will be needed for better assessment of pulmonary findings and other skeletal anomalies.

## Learning Points

1. MRKH syndrome refers to a rare congenital condition characterized by aplasia of the uterus and the upper part (two-thirds) of the vagina. It presents with primary amenorrhoea in females with normal ovaries and fallopian tubes, secondary sexual characteristics and 46XX karyotype.
2. MRKH syndrome is associated with wide-ranging renal, skeletal, hearing, cardiac and pulmonary anomalies.
3. Pulmonary anamolies are rare features of MRKH syndrome and could include complete absence of the lung and the bronchus with no vascular supply to the affected side, a rudimentary bronchus with complete absence of the pulmonary parenchyma or a variable amount of bronchial tree, pulmonary parenchyma and supporting vasculature.
4. CT scan is a better tool for assessment of the lung parenchyma, tracheobronchial tree and skeletal anomalies.
5. MRI on the other hand is a very good non-invasive technique for assessing the pelvic organs, spinal cord, disc and osseous structures. *T*_2_ weighted MRI sequences produce excellent zonal anatomy of the uterus, including the endometrium, junctional zone and myometrial anatomy.
